# Tracing CO_2_ emissions across megacity landscapes: beyond citywide totals to structural heterogeneity and mitigation

**DOI:** 10.1016/j.ese.2025.100602

**Published:** 2025-07-12

**Authors:** Yiwen Zhu, Yuhang Zhang, Yi Zhang, Bo Zheng

**Affiliations:** a Shenzhen Key Laboratory of Ecological Remediation and Carbon Sequestration, Institute of Environment and Ecology, Tsinghua Shenzhen International Graduate School, Tsinghua University, Shenzhen, 518055, China; b State Environmental Protection Key Laboratory of Sources and Control of Air Pollution Complex, Beijing, 100084, China; c Institute of Future Human Habitats, Tsinghua Shenzhen International Graduate School, Tsinghua University, Shenzhen, 518055, China

**Keywords:** CO_2_ emissions, Structural heterogeneity, Scope 1, Scope 2, Megacity, Targeted reduction strategies

## Abstract

Cities are central to global climate change mitigation efforts due to their substantial carbon emissions. Effective, evidence-based climate policy requires a detailed understanding of urban carbon metabolism, allowing for targeted mitigation pathways and the accurate evaluation of sustainability. However, a persistent lack of clarity on how carbon flows are distributed spatially and sectorally within cities has hindered tailored climate action, particularly in rapidly developing megacities. Here we map the shifting landscape of carbon emissions in Chinese megacities and show that accountability for these emissions has undergone a profound spatial and sectoral transformation. We found that the primary burden of emission responsibility has moved from production-focused sectors, such as industry and energy generation, to consumption-based end-users, including residential and commercial buildings. This transition is driven by a structural shift in accounting boundaries from direct fossil fuel combustion (Scope 1) to indirect emissions from electricity consumption (Scope 2), fundamentally redistributing carbon liability across urban districts. Our landscape-level framework reveals the hidden carbon dependencies of end-use sectors and provides a model for equitable and effective accounting, enabling the design of region-specific strategies to address the complexities of urban carbon emissions.

## Highlights

• This study depicts the point/street/building-level distribution patterns of CO_2_ emissions within a megacity in China.
• Our results illustrate structural heterogeneity in emission mitigation potential between Scope 1 and Scope 2.
• Our study reveals carbon flows within megacity and provides insights into targeted reduction strategies.

## Graphical abstract

**Figure:**
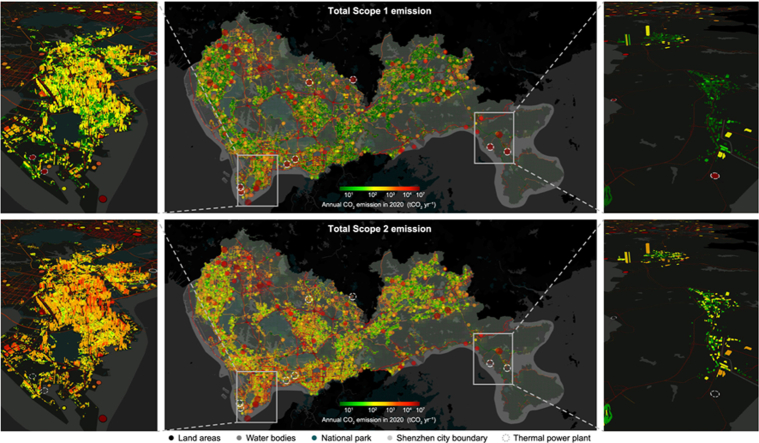

## 1 Introduction

Cities, home to over 55 % of the global population, contribute more than 70 % of global fossil fuel carbon dioxide (CO_2_) emissions, rendering them the largest anthropogenic contributor to climate change [[1], [2], [3]]. However, as centers of economic activity and energy consumption, cities are not only major CO_2_ emitters but also possess the resources and tools necessary to confront climate challenges [2,4,5]. Detailed and accurate CO_2_ emission accounting approaches for cities are both crucial and urgent for designing emission reduction measures [6,7] and evaluating the effectiveness of mitigation efforts. Among cities of different scales, megacities (i.e., metropolitan areas with populations exceeding 10 million) are characterized by high carbon emission intensities, making them hotspots for urban CO_2_ emissions monitoring and mitigation [8,9]. China, as one of the most populous countries, has several megacities with dense economic activities and large populations. With the acceleration of urbanization and ongoing economic growth in China, the anticipated increase in CO_2_ emissions from megacities poses a threat to China in meeting its national emission reduction targets, which is also a challenge to global climate goals [10]. Investigating the CO_2_ emission characteristics of Chinese megacities is essential for advancing actions to reduce greenhouse gas emissions and manage climate risks.

High-resolution carbon emissions mapping that identifies emission hotspots and metabolic pathways within megacities provides valuable datasets for designing and implementing effective mitigation policies. In addition to mapping total emissions, a concerted effort is needed to enhance the spatial granularity of emissions mapping for the urban landscape scale and to illustrate detailed emission characteristics, supporting accurate and timely management strategies for cities [11]. Previous studies on urban emissions mapping have advanced toward finer granularity. For example, Gurney et al. [12] demonstrated how the distribution of fossil fuel CO_2_ emissions in Indianapolis at the building and street levels could provide new perspectives for monitoring and managing urban-scale emissions. For the road transportation sector in the United States, Gately et al. [13,14]. developed the Road Transportation Emissions Database, which estimated on-road CO_2_ emissions at a 1 × 1 km^2^ resolution from 1980 to 2012. Moran et al. [15] accounted for CO_2_ emissions across 108,000 European cities, allocating emissions to registered buildings and vehicle fueling stations based on OpenStreetMap. However, these studies focused on the United States and Europe, while megacities in China still lack high-resolution emissions mapping.

In China, city-level emissions accounting has mostly focused on annual emission totals [[16], [17], [18]] and, in some case studies, hourly timescales [19]. Such inventories simplify or overlook the detailed emission distributions within city boundaries, as they aim to determine temporal trends in emissions and analyze drivers for cities. Previous studies have spatially downscaled emissions for individual or several sectors in China's cities [[20], [21], [22]]. However, these studies were based on spatial proxies (e.g., population density, road density, or nighttime light) [23], with substantial uncertainties at the fine scale [24,25]. Given the spatial heterogeneity in industrial facilities, traffic flow, and building distributions, it is crucial to depict carbon emission flows or carbon metabolism within megacities. Such information can support emissions monitoring and effective strategies for sustainable urban development and CO_2_ emission reductions. In addition, most current emission inventories focus on Scope 1 emissions [19,26] (i.e., direct emissions resulting from human activities occurring within the city boundary [27]), while few studies have reported Scope 2 emissions (i.e., indirect emissions involving imported electricity consumption [28]) or have done so without reflecting their spatial distributions [29,30].

**Fig. 1:**
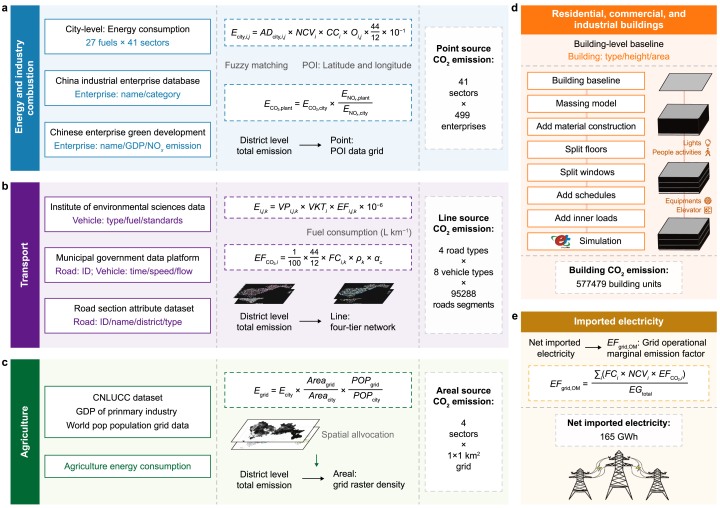
The CO_2_ emission accounting framework for megacities of Shenzhen. **a**, Energy and industrial point sources; **b**, Transport line sources; **c**, Agricultural areal sources; **d**, Residential, commercial, and industrial buildings; **e**, Imported electricity. The Scope 1 emission accounting (panels **a**–**d**) utilizes city-level energy consumption as a comprehensive constraint, followed by the input dataset and spatial and sectoral allocation methodology for each sector. Additionally, panel **d** illustrates the simulation process of operating energy consumption for individual buildings, which is employed to map emissions associated with imported electricity (panel **e**) under the Scope 2 accounting framework. GDP: gross domestic product; POI: points of interest.

This study focused on the abovementioned challenges by accounting for CO_2_ emissions at the megacity landscape level and investigating the metabolic processes of carbon emissions within the megacity of Shenzhen, China. Integrating factory-level information, land-use patterns, traffic flows, points of interest, and individual building energy consumption simulation, we developed a comprehensive urban emissions accounting model under the Scope 1 and Scope 2 frameworks (Fig. 1, see the Materials and Methods section). The model was applied to Shenzhen—China's earliest special economic zone and fastest urbanizing city [31]—to account for and track carbon emission flows across different fuels, sectors, industrial infrastructures, road networks, and buildings. We analyzed the spatial relationship between the distribution of Scope 1 and Scope 2 CO_2_ emissions and population distribution, providing database support for monitoring urban carbon budgets, developing carbon mitigation strategies, and assessing the effectiveness of emission reductions at the city level, with potential applications to other megacities.

## 2 Materials and methods

### 2.1 General methodology framework

CO_2_ emissions are usually accounted for based on energy balance statistics, which include the consumption of various types of fuels within the statistical boundary. This conventional approach has been widely used for direct emissions estimation under Scope 1. However, it excludes the indirect emissions associated with electricity imports under Scope 2. To address this gap, we developed a framework to estimate both Scope 1 and Scope 2 CO_2_ emissions at the city level by sector and fuel (Fig. 1). For Scope 1 emissions, we adopted Shan et al.‘s [32] methodology to extend the provincial energy balance statistics to construct city-level energy balance tables, tracking the consumption of 27 types of fuels across 47 socioeconomic sectors. For the emission factors, we utilized the corrected values from Liu et al. [33] based on empirical measurements of fossil fuels in China, calculating them based on the carbon content (*CC*), net caloric value (*NCV*), and oxidation efficiency (*O*) of fossil fuels. The final CO_2_ emissions by economic sector and fuel type were calculated based on equation (1) [34].

(1) $$E_{\text{city},i,j}={AD}_{c\text{ity},i,j}\times {NCV}_{i,j}\times {CC}_{i}\times {\;O}_{i,j}\times \frac{44}{12}\times 10^{-1}$$

where *i* and *j* are the indices representing fossil fuel type and sector, respectively. *i* represents the various types of fossil fuels within China's energy statistical system, totaling 27; *j* represents 47 socioeconomic sectors. *E*_city*,i,j*_ (t CO_2_) represents city-level CO_2_ emissions of fossil fuel *i* from sector *j*. *AD*_city*,i,j*_ (t for solid fuels or 10^8^ m^3^ for gases) denotes city-level activity data corresponding to the consumption of fossil fuel *i* in sector *j*; *NCV*_*i,j*_ indicates the net calorific value for fuel *i* in sector *j* (PJ per 10^4^ t for solid fuels or PJ per 10^8^ m^3^ for gases); *CC*_*i*_ indicates the carbon content of fuel *i* (t C TJ^−1^); and *O*_*i,j*_ represents the combustion oxidation rate, indicating the extent to which fuel is oxidized during combustion (in units of %).

For Scope 2 emissions, we estimated imported electricity-related emissions and spatially allocated them to residential, commercial, and industrial buildings based on building-level energy consumption. The CO_2_ emissions responsibility associated with imported electricity was reallocated from power plants outside the city to within the city boundary under the Scope 2 accounting framework. In this study, net imported electricity was used to represent this shift in emissions responsibility, with the CO_2_ emission intensity of imported electricity calculated based on the grid operational marginal emission factor (*EF*_grid,OM_) for the external power grid supplying the city, based on equation (2). Our framework also considered the spatial and sectoral redistribution of emissions due to electricity consumption within the city boundary. Scope 1 emissions from local power generation, initially assigned to power plant point sources, were reallocated to end consumption under the Scope 2 framework, which includes residential, commercial, and industrial buildings.

(2) $${EF}_{g\text{rid},\text{OM}}=\frac{\sum_{i\;}\left(\;{FC}_{i}\times {NCV}_{i}\times {EF}_{{\text{CO}}_{2},i}\right)}{{EG}_{t\text{otal}}}$$

where *i* represents the types of fossil fuels consumed by the power system for electricity generation; *EF*_grid,OM_ (tCO_2_ MWh^−1^) represents the simple marginal CO_2_ emission’ factor of the grid operational electricity generation; *FC*_*i*_ (10^4^ t or 10^8^ m^3^) is the total fuel consumption of fuel *i* by the power system; *NCV*_*i*_ (GJ per 10^4^ t or 10^8^ m^3^) is the average lower heating value of fuel *i*; ${EF}_{{\text{CO}}_{2},i}$ (tCO_2_ GJ^−1^) is the CO_2_ emission factor of fuel *i*; and *EG*_total_ (MWh) is the total net electricity generation of the power system.

### 2.2 Essential data input

Integrating both Scope 1 and Scope 2 sectoral carbon emissions, this accounting framework has been developed with scalability for potential application in global megacities with similar development contexts and data foundations. The essential input data sources include both city- and sectoral-level information. At the city level, the primary data sources include energy consumption to ensure the accuracy of total emissions estimates, specifically fossil fuel consumption by industry, total electricity generation, and electricity usage. At the sectoral level, the data required involve the following intensity profiles for carbon emission-related processes: (a) for the energy and industry combustion sector, factory locations and fuel consumption data associated with industry-level totals, in which factory-specific nitrogen oxide (NO_*x*_) emissions data can serve as CO_2_ emission proxies if specific fuel utilization data are unavailable (e.g., in the case of Shenzhen in this study); (b) for transportation, vehicle fleet composition, road location and type, and information on the implementation of emission control standards, while different resolutions of traffic flow data are optional and are helpful to enhance the spatial resolution of emissions mapping; (c) for residential, commercial, and industrial buildings, building location, building standards, and local weather conditions (to model the electricity consumption of individual buildings); and (d) for agriculture, forestry, livestock, and fisheries, land use and rural population distribution.

This multiperspective methodology framework is demonstrated through a case study of Shenzhen. Fossil energy consumption by sector was derived from the provincial energy balance tables, enabling the calculation of Scope 1 direct emissions. Under the Scope 2 accounting framework, all electricity imported from outside Shenzhen in 2020 (16.5 million MWh) was assumed to originate from the Southern Power Grid, with 55.6 % of the imported electricity generated from clean energy sources (http://www.sasac.gov.cn/n2588025/n13790238/n14546245/c19584965/content.html). Although this clean energy proportion is lower than that in Shenzhen's local electricity generation mix, it is substantially higher than the national average level of 43.4 % (https://www.nea.gov.cn/2021-01/20/c_139683739.htm). Taking into account the clean energy structure of the Southern Power Grid, we utilized equation (2) to calculate the *EF*_grid,OM_, which was determined to be 0.433 kg CO_2_ kWh^−1^. The indirect CO_2_ emissions associated with imported electricity were then calculated by multiplying the net imported electricity by this emissions factor.

### 2.3 Energy and industrial combustion sources

City-level energy consumption data encompassed 27 types of fossil fuels across 41 industrial subsectors, providing a top-level constraint on emissions from energy and industrial combustion sources. To obtain detailed enterprise-level information, we integrated the China Industrial Enterprise Database and the Chinese Enterprise Green Development Database based on enterprise codes. These two databases contain critical information, including industry classification, NO_*x*_ emissions, and economic production. The CO_2_ emissions were distributed across enterprises within the same sector based on the NO_*x*_ emission percentages using equation (3), considering the coemissions of CO_2_ and NO_*x*_. We further employed the FuzzyWuzzy library from Python to conduct fuzzy matching of enterprise names with industrial points of interest, enabling the determination of geographic coordinates for 499 enterprises.

(3) $$E_{{\text{CO}}_{2},\text{plan}t,j}=E_{{\text{CO}}_{2},\text{cit}y,j}\times \frac{E_{{\text{NO}}_{x},\text{plan}t,j}}{E_{{\text{NO}}_{x},\text{cit}y,j}}$$

where $E_{CO_{2},\text{city},j}$ and $E_{CO_{2},\text{plant},j}$ represent the CO_2_ emissions from energy and industrial sector *j* at the city and enterprise levels, respectively. $E_{{\text{NO}}_{x},\text{city},j}$ and $E_{{\text{NO}}_{x},\text{plant},j}$ represent the NO_x_ emissions from sector *j* at the city and enterprise levels, respectively.

Under the Scope 2 accounting framework, we excluded direct emissions from fuel combustion by power generation enterprises from the energy and industrial combustion sources and reallocated them to residential, commercial, and industrial buildings based on the reconstruction of individual building electricity consumption.

### 2.4 On-road transportation sources

The on-road transportation emissions from fuel combustion were estimated using the city-level vehicle emission model developed in our previous work [35], briefly shown in equation (4) (Supplementary Fig. S1). We applied localized emission factor corrections based on traffic flow data, including driving speed, road IDs, traffic volume, and time, sourced from the Shenzhen Municipal Government Data Platform (https://opendata.sz.gov.cn/). By incorporating road-level traffic information in the city-level vehicle emission model, we mapped activity data and emission factors to better reflect local driving behavior.

(4) $$E_{m,n,k}={VP}_{m,k}\times {\;VKT}_{m,n}\times {\;EF}_{m,n,k}\times 10^{-6}$$

where *m* represents vehicle type, including four passenger vehicle types: heavy-duty buses, medium-duty buses, light-duty buses (including cars), and mini-duty buses; and four truck types, namely, heavy-duty trucks, medium-duty trucks, light-duty trucks, and mini-duty trucks. *n* represents road type, including first-level roads (e.g., highways and urban expressways), second-level roads (e.g., national and provincial roads), third-level roads (e.g., county roads), and fourth-level roads (e.g., intra-urban and rural roads). *k* represents the fuel type, including diesel and gasoline. *E*_*m,n,k*_ (t CO_2_) represents the annual CO_2_ emissions of vehicle type *m* on road type *n* using fuel type *k*. *VP*_*m,k*_ refers to the number of vehicles of type *m* using fuel type *k*. *VKT*_*m,n*_ (km year^−1^) is the average mileage traveled per year of vehicle type *m* on road type *n*. *EF*_*m,n,k*_ (g km^−1^) is the emission factor for vehicle type *m* on road type *n* using fuel type *k*.

Vehicle ownership (*VP*) data from the Shenzhen Institute of Environmental Sciences and the Shenzhen Municipal Bureau of Statistics (https://tjj.sz.gov.cn/) were used to construct a vehicle fleet distribution model, covering the eight vehicle types described above. For the emission factors (*EF*s), we utilized road-level vehicle speed information and applied speed-related fuel consumption corrections to the EF adjustment, reflecting the impact of different driving behaviors on emissions (Supplementary Fig. S1). First, we extracted the average vehicle speed for each district. To control data quality, vehicle speeds within one standard deviation were selected for the calculation of the weighted average speed. Based on the average speed, we used the vehicle speed–fuel consumption curves corresponding to different vehicle types and road types to reconstruct the road segment-level fuel consumption (*FC)* and calculated the localized *EF* using tary equation (5).

(5) $${EF}_{m,n,k}=\frac{1\;}{100}\times \frac{44}{12}\times {\;FC}_{m,n,k}\times {\;\rho }_{k}\times \alpha$$

where *EF*_*m,n,k*_ (g km^−1^) indicates the CO_2_ emission factor for vehicle type *m* on road type *n* using fuel type *k*; *FC*_*m,n,k*_ (L per 100 km) represents the consumption of fuel type *k* for vehicle type *m* on road type *n*; *ρ*_*k*_ *(*g L^−1^) is the density of fuel type *k*, whereby gasoline is 770 and diesel is 840; and *α* is the mass fraction of carbon, based on the value of 0.87 in Wen et al.‘s study [20].

Spatially, the traffic flow data were matched with the Road Section Attribute Dataset using road IDs to identify the administrative district and road type associated with each road segment, enabling the allocation of traffic volume across districts. Finally, based on the high-resolution, localized vehicle emission model, CO_2_ emissions from Shenzhen's on-road transportation for the year 2020 were presented as line sources for 95,288 road segments, covering 8 vehicle types and 4 road levels.

### 2.5 Residential, commercial, and industrial buildings

Simulating the operational conditions of individual buildings and the associated annual electricity consumption is a crucial step in the spatial distribution of Scope 2 emissions. To accurately characterize electricity consumption patterns in individual buildings, we developed a scalable, bottom-up modeling framework for simulating annual operational energy use in urban building clusters. In this framework, Ladybug Tools was used as an extensive interface, linking three-dimensional (3D) computer-aided design with validated simulation engines for diverse applications (https://www ladybug tools). In this study, it was utilized to generate 3D building models, including envelopes and equipped systems. This tool enables the construction of building models based on physical parameters and schedules, such as occupancy and device usage. It supports both physical and economic analyses through various simulation engines. For our building load simulations, EnergyPlus was chosen due to its superior performance compared to other engines [36] and its widespread adoption in this field [[37], [38], [39]]. The building geometries were then transformed into building blocks based on their height. Wall, floor, and window building materials were then added to the building blocks, which were segmented into multiple floors, with windows added according to predefined window-to-wall ratios. Occupancy schedules and device operation schedules were set based on the building type. Inner parameters, such as device energy consumption densities, were also defined. Finally, the constructed building model was saved as an OpenStudio Workflow file and used as input for the EnergyPlus simulations.

For Shenzhen, the generation process (Fig. 1d) began with acquiring geometric data for 577,480 individual building units from AMap (https://www.amap.com), followed by applying region-specific inputs used in building load generation following Shenzhen's local standards. Building materials conformed to the ASHRAE 198.1–2010 standard, which provides a material library tailored to different climate zones (https://www.ashrae.org). Each floor height was assumed to be 3 m, according to the AMap data. The window-to-wall ratios were determined according to the Code for Thermal Design of Civil Buildings [40]. Occupancy and device operation schedules were detailed based on the Department of Energy guidelines from 2011 [41]. Inner building parameters were selected following the Standard for Green Performance Calculation of Civil Buildings 2018 (JGJ/T449-2018, https://www.mohurd.gov.cn). Weather data for the EnergyPlus simulations were derived from Climate.OneBuilding.Org (climate.onebuilding.org), providing meteorological data spanning 2007–2021. Building types were identified using land planning information from the Shenzhen Municipal Bureau of Planning and Natural Resources (http://pnr.sz.gov.cn), which categorizes areas within the city by function, aiding building type determination for the assignment of appropriate schedules and parameters. EnergyPlus outputs hourly building energy consumption profiles for an entire year. This integrated approach, utilizing Ladybug Tools and EnergyPlus, facilitated robust building load simulations, ensuring that the constructed models accurately reflected real-world conditions and enabling informed decision-making in building design and energy management.

### 2.6 Agriculture, forestry, livestock, and fisheries sources

This source sector corresponds to direct emissions from fossil fuel combustion related to agriculture, forestry, livestock, and fisheries activities. Ten land-use types, including paddy fields, drylands, shrubs, and reservoirs, were identified using the land-use classification distribution map provided by the CNLUCC Dataset (http://www.resdc.cn/DOI). We further assumed that these anthropogenic emissions were associated with population distribution. Therefore, the spatial distribution of land-use types was unified with the WorldPop Population Data (https://hub.worldpop.org/) into a 1 × 1 km^2^ grid for emission mapping to grid cells.

## 3 Results

### 3.1 Metabolic processes underlying Shenzhen's CO_2_ emissions

The anthropogenic CO_2_ emissions from Shenzhen were estimated to be 52.0 Tg CO_2_ yr^−1^ in 2020 based on the Scope 2 framework. A total of 86.3 % of these emissions, equivalent to 44.8 Tg CO_2_ yr^−1^, were directly released from local sources within the Shenzhen boundary (Scope 1 estimate), and the remaining 13.7 % (7.2 Tg CO_2_ yr^−1^) were associated with electricity imported across the city border, which was consumed by Shenzhen's industries and residents but generated outside of and transferred to the city. The amount of imported electricity was calculated based on the difference between electricity consumption and generation in Shenzhen, representing the net imported amount. The estimated direct emissions are comparable to the estimate by the Carbon Emission Accounts and Datasets (CEADs) [16,26,32,42] which reported 44.3 Tg CO_2_ yr^−1^ for Shenzhen in 2019 (https://www.ceads.net.cn/). According to the CEADs data, Shenzhen has the lowest CO_2_ emissions among the six megacities with a population of over 15 million in China (ranging from 44.3 to 192.9 Tg CO_2_ yr^−1^).

**Fig. 2:**
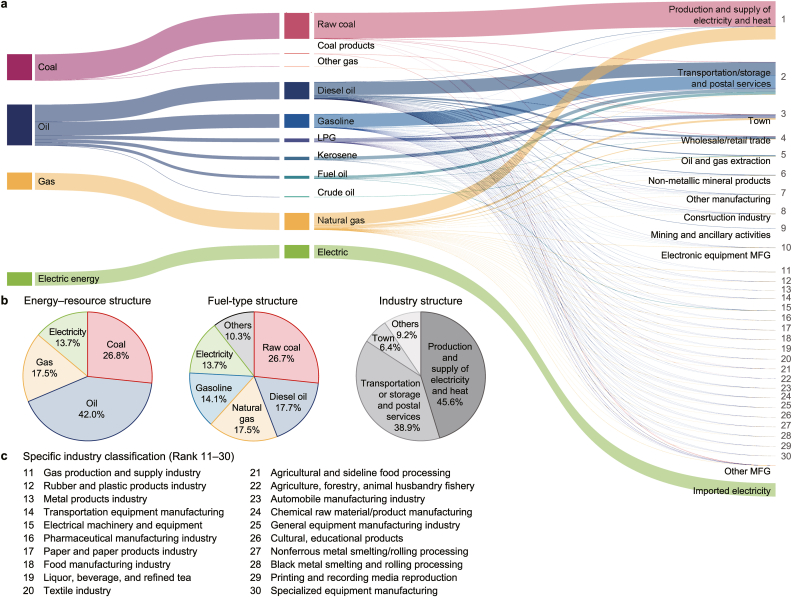
Emission metabolic pathway of Shenzhen city. **a**, Carbon emission flow within the city among fuel types and sectors, with the thickness of the flow representing the amount of CO_2_ emissions. Numbers: Serial numbers of the top 30 carbon-emitting industries. LPG: Liquefied Petroleum Gas. MFG: Manufacturing. **b**, The emission structure by energy, fuel type, and specific industries. **c**, The specific names of industries outside the top ten emitting sectors.

Shenzhen's emission characteristics are rooted in its energy and economic structures, with oil, coal, and natural gas contributing 42.0 %, 26.8 %, and 17.5 %, respectively, to the city's total CO_2_ emissions in 2020 (Fig. 2). These three types of fossil fuel accounted for nearly half of Shenzhen's primary energy consumption in 2020, with their shares being 28.4 %, 11.4 %, and 12.7 %, respectively (https://meeb.sz.gov.cn/). The remaining half of Shenzhen's primary energy consumption was supplied by primary electricity sources, including nuclear power, hydropower, and solar power, as well as imported electricity. The high proportion of primary electricity and natural gas in Shenzhen's energy consumption has gradually reduced its reliance on coal and oil, resulting in a 4.5 % decrease in their usage from 2015 to 2020. Shenzhen's low coal-dependent energy structure has led to a lower proportion of coal-related emissions (26.8 %), in stark contrast to the top 10 highest-emitting cities nationwide, which have an average of 81.9 % coal in their emission structures (ranging from 53.7 % to 96.9 %) [26].

The thermal power plants in Shenzhen generated 20.4 Tg CO_2_ yr^−1^ in 2020, making it the largest single emissions source (39.3 %), with coal and natural gas contributing 66.6 % and 33.4 % of the related emissions, respectively. By fuel type, thermal power plants were the largest contributors to coal (97.9 %) and natural gas (75.0 %) emissions in the city; however, they were not the dominant electricity generators. In 2020, Shenzhen's total electricity generation reached 81.8 million MWh, with clean energy sources accounting for 69.3 % of production. The installed capacity of non-fossil fuel sources—which produce no direct carbon emissions—comprised wind power (2884.1 MW), solar photovoltaic power (1062 MW), hydropower (991.5 MW), waste-to-energy (719.5 MW), and other generation facilities.

This shows Shenzhen's sustained progress toward a low-carbon transformation of the city's power generation structure. Since the carbon intensity of natural gas power generation is half that of coal, combined with the high proportion of non-fossil energy, the carbon intensity of Shenzhen's power generation was calculated at 0.25 kg CO_2_ kWh^−1^ in 2020, lower than half of the national grid average of 0.56 kg CO_2_ kWh^−1^ (https://www.gov.cn/lianbo/bumen/202404/content_6945445.htm).

The transportation sector released 17.4 Tg CO_2_ yr^−1^ in 2020, making it the second-largest source of emissions in Shenzhen (38.9 %) and the largest contributor to oil-related emissions (79.5 %). Emissions from this sector comprised gasoline (40.6 %), diesel (39.9 %), kerosene (10.2 %), and other sources (9.3 %). The high oil consumption was primarily due to the large number of civilian vehicles, which steadily increased by 12.3 % from 2015 (3.1 million) to 2020 (3.5 million). Despite covering only 1.1 % of Guangdong Province's land area, Shenzhen accounted for 14.1 % of the province's total number of civil vehicles. Among the vehicle types, light-duty buses (including private cars) and heavy-duty trucks were the primary contributors to gasoline emissions (88.0 %) and diesel emissions (73.9 %), respectively. To control transportation emissions, Shenzhen has promoted the adoption of electric vehicles. We assumed that all charging processes occur at buildings, so the associated emissions are accounted for in residential, commercial, and industrial buildings rather than in the transportation sector.

From the Scope 1 perspective, the combustion sources related to residential, commercial, and industrial buildings emitted 5.0 Tg CO_2_ yr^−1^, making it the third-largest emission source. However, allocating the electricity generation emissions at the location of electricity consumption under the Scope 2 perspective, emissions from residential, commercial, and industrial buildings increased to 32.6 Tg CO_2_ yr^−1^, about six times higher than the Scope 1-based estimate. The increase of 27.6 Tg CO_2_ is due to two reasons: (a) local electricity emissions produced by local power plants within the city accounted for 20.4 Tg CO_2_, and (b) external electricity emissions associated with electricity generated outside Shenzhen accounted for 7.2 Tg CO_2_. Residential, commercial, and industrial buildings emerged as the largest contributors to the Scope 2-based emissions estimate (62.6 %), consisting of local electricity emissions (62.8 % of the sector), external electricity emissions (21.9 % of the sector), and Scope 1 emissions (15.3 % of the sector). We allocated these emissions to individual buildings based on their simulated annual electricity consumption, which highlights the substantial differences in emissions burden and responsibility between power generation and consumption for developing city-scale carbon emission reduction strategies.

### 3.2 Mapping CO_2_ emissions across megacity landscapes

**Fig. 3:**
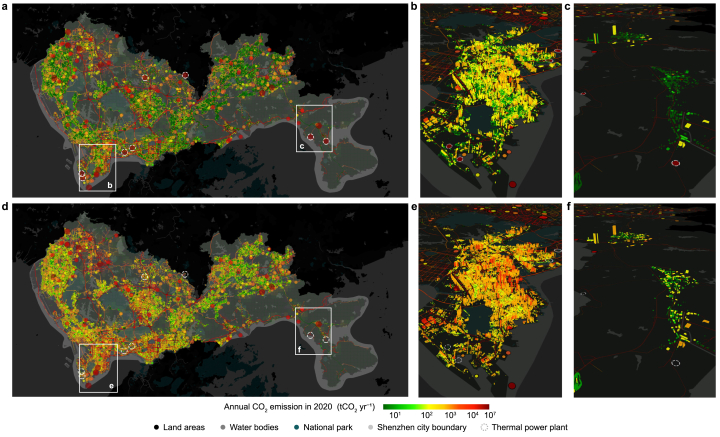
The spatial distribution of Scope 1 (**a**) and Scope 2 (**d**)-based CO_2_ emissions in Shenzhen in 2020 with Nanshan District (**b**, **e**) and Dapeng District (**c**, **f**) by partial enlargement.

**Fig. 4:**
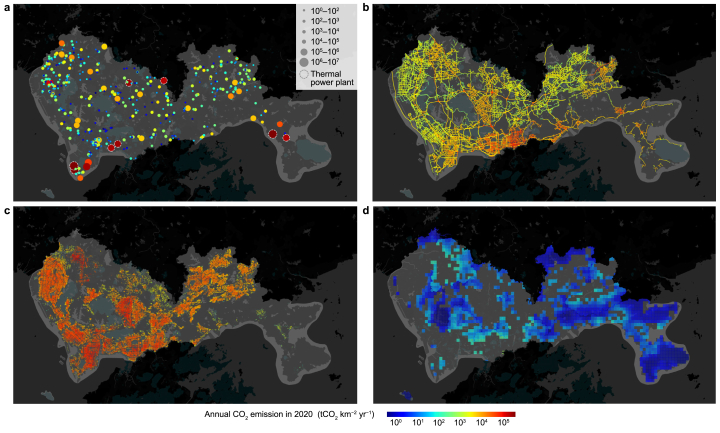
Spatial distribution of Scope 2-based CO_2_ emissions by sector in Shenzhen in 2020. The CO_2_ emission maps include: **a**, energy and industry combustion sources; **b**, on-road transportation sources; **c**, residential, commercial, and industrial buildings; **d**, other sources, including agriculture, animal husbandry, fisheries, and forestry. Point sources in panel **a** include thermal power plants (dashed circle) and other plants (solid circle). The other emission maps (panels **b**–**d**) have a spatial resolution of 1 × 1 km^2^.

We mapped the Scope 1 and Scope 2 estimates of CO_2_ emissions for Shenzhen (Fig. 3, Fig. 4) across multiple spatial granularities: point, line, area (1 × 1 km^2^ grids), and individual buildings. The point sources encompass the combustion facilities for energy and industrial production, and the line sources represent direct emissions from vehicles on road networks. The residential, commercial, and industrial building sources include direct combustion (Scope 1) and indirect electricity-related (Scope 2) emissions that occur within and related to individual buildings. Area sources include combustion emissions from agriculture, forestry, livestock, and fisheries sources based on the corresponding land-use type. Our results show that the point (50.0 %) and line sources (38.9 %) dominated Scope 1 emissions (88.9 %), while residential, commercial, and industrial buildings dominated Scope 2 emissions (62.6 %). The shifts in the spatial mapping of emissions between Scope 1 and Scope 2 estimates reveal the uneven distribution of emissions across hotspot areas and sectors.

The distribution of Scope 1 emissions exhibited spatial heterogeneity, with emission hotspots predominantly located around major point and line sources. The main emission point source is the eight thermal power plants (shown in the white dashed circles in Fig. 3a and b), which contribute 91.2 % of total point-source emissions. The high and concentrated emissions from thermal power plants resulted in a highly uneven spatial distribution, with only 0.3 % of the land areas contributing to 45.6 % of Scope 1 emissions. Emission hotspots from line sources are concentrated in the central-southern regions of Shenzhen (Fig. 4b), particularly in Luohu District (31.9 Gg CO_2_ km^−2^, as detailed in Supplementary Fig. S2 and Table S1) and Futian District (28.1 Gg CO_2_ km^−2^), with line source emission intensities in both districts substantially exceeding the city's average level for line sources (8.7 Gg CO_2_ km^−2^). In Luohu and Futian districts, intra-urban roads serve as the major contributors to line emissions, accounting for 56.0 % and 72.9 %, respectively. Despite representing only 11.5 % of the total length of intra-urban roads in Shenzhen, Luohu and Futian Districts are responsible for 36.4 % of the total emissions from these roads due to their dense economic activities associated with a high concentration of traffic flow. These line source emissions are decoupled from road length, demonstrating that this study, by integrating traffic flow data, reduced dependence on road length as the sole proxy for allocating road emissions.

The distribution of Scope 2 emissions exhibited a reduced spatial heterogeneity but revealed a sectoral imbalance due to the allocation of emissions to buildings. The point source emissions decreased from 22.4 Tg CO_2_ yr^−1^ (50.0 %) to 2.0 Tg CO_2_ yr^−1^ (3.8 %), while the residential, commercial, and industrial building emissions increased from 5.0 Tg CO_2_ yr^−1^ (11.1 %) to 32.6 Tg CO_2_ yr^−1^ (62.6 %) (Fig. 4a–d). Such a disparity is due to the spatial shift of emissions from 8 thermal power plants to 577,480 buildings, which corresponds to the transition of electricity emissions from production locations to end consumption under the Scope 2 framework. Despite the intensified sectoral imbalance, the spatial clustering effect has been mitigated. For example, for the hotspot grid areas with 50 % of total emissions (ranked in descending order of 1 × 1 km^2^ grid emissions), the total land areas within Shenzhen expand from 0.7 % in Scope 1–12.7 % in Scope 2. This expansion of high-emission areas reveals a reduction in the uneven distribution of emissions within the city and the broad emission mitigation responsibility on the consumption side under the Scope 2 accounting framework.

The emission distribution patterns correspond to the functional designations of different districts within Shenzhen. The districts with a decrease in Scope 2 emissions are the electricity production-led areas, including Nanshan and Dapeng districts. Nanshan District, which is dominated by residential, commercial, and industrial buildings (68.9 %), had the largest Scope 1 emission of 19.8 Tg CO_2_ yr^−1^, primarily driven by point sources (87.1 %), and experienced a decrease in Scope 2 emissions to 8.2 Tg CO_2_ yr^−1^. The increase in emissions from the building sector (as shown in Fig. 3c and d) did not offset the reduction in emissions from power generation, such as emissions from Mawan Power Co., Ltd., the largest thermal power plant in Shenzhen. Dapeng District has the lowest Scope 2 emissions in Shenzhen despite an increase in emissions (Fig. 3e and f). This is consistent with Dapeng District's functional designation as a coastal ecological tourism and resort area, where forest coverage exceeds 77.0 % (Fig. 4d), resulting in lower emissions due to limited human activities. In contrast, Baoan District had the highest electricity consumption in 2020 (24.4 million MWh) but no thermal power plants. This resulted in this district having the largest Scope 2 emissions (10.7 Tg CO_2_ yr^−1^), with only 3.9 Tg CO_2_ yr^−1^ Scope 1 emissions. This difference aligns with Baoan's role as a center of economic agglomeration, exerting potential radial influence within Shenzhen.

### 3.3 Heterogeneity in emission distributions across population densities

**Fig. 5:**
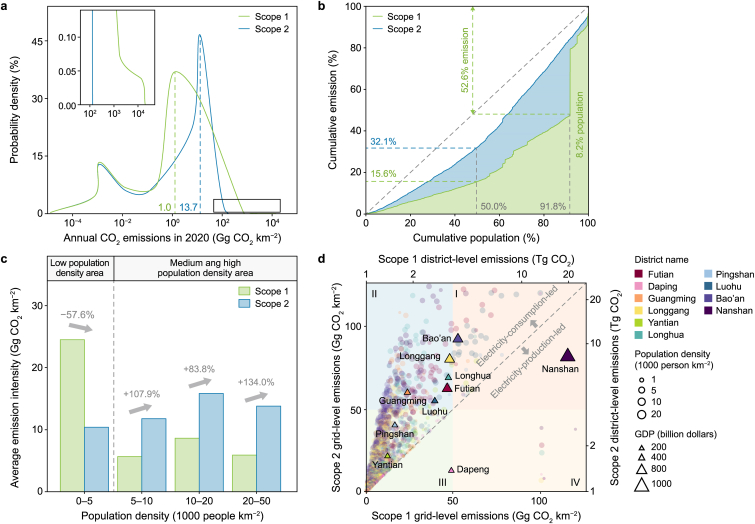
Comparison of emission distribution patterns in Shenzhen between Scope 1 and Scope 2-based estimates. The emission distribution maps are processed to a spatial resolution of 1 × 1 km^2^. **a**, The probability distribution of gridded emissions from all grid cells, with an inserted figure showing the enlarged view of super-emission areas (exceeding 10^3^ Gg CO_2_ km^−2^). **b**, Emission–Lorentz curve of descending population density across grid cells. The dashed line represents the y = x line. **c**, The variation in gridded emissions across areas of different population densities. **d**, Identification of emission hotspots at grid (circles) and district (triangles) levels. Region I: High Scope 1 & 2 emissions; Region II: Higher Scope 2 than Scope 1; Region III: Low Scope 1 & 2 emissions; Region IV: Higher Scope 1 than Scope 2.

The spatial distributions of Scope 1 and Scope 2 emissions in Shenzhen align with low-emission areas (less than 10^−1^ Gg CO_2_ km^−2^) but diverge over high-emission areas (ranging from 10^−1^–10^4^ Gg CO_2_ km^−2^) (Fig. 5a). Total emissions were aggregated into 1 × 1 km^2^ grids, and the frequency analysis revealed a bimodal distribution for both estimates (Fig. 5a). The first peak was observed over low-emission areas, with the agricultural sector as the primary contributor, accounting for 84.5 % and 73.7 % of the corresponding total emissions, respectively. The second peak was observed over high-emission areas, revealing differences in both peak emission intensity and distribution frequency between Scope 1 (1.0 Gg CO_2_ km^−2^, 30.4 %) and Scope 2 (13.7 Gg CO_2_ km^−2^, 46.2 %) estimates. The enhancement and expansion of emissions in the Scope 2 estimates coincide with the reallocation of emissions from electricity generation, showing a small peak over super-emission areas (exceeding 10^3^ Gg CO_2_ km^−2^) in the Scope 1 estimates (Fig. 5a).

The Scope 1 and Scope 2 emissions both exhibit a nonlinear relationship with population density (Fig. 5b). By ranking grid cells according to descending population density, the Lorenz curves for emission estimates form concave distributions below the *y* = *x* line (Fig. 5b), reflecting an uneven distribution of emissions relative to the population. The Gini coefficient was calculated to quantify this inequality, with higher values indicating greater unevenness in the distribution of population and emissions. The Scope 2 emissions exhibit a more even distribution relative to population density (Gini coefficient = 0.4) compared to the Scope 1 emissions (Gini coefficient = 0.7). The Scope 2 curve's slope, which is closer to 1, indicates that emissions redistributed based on electricity consumption in buildings are spatially closely associated with human activity. This is further reflected in the top 50 % of the cumulative population areas in the Lorenz curve, where the corresponding cumulative emissions account for a higher percentage in Scope 2 (32.1 %) compared to Scope 1 (15.6 %). The steeper Scope 1 curve in low-population-density areas leads to substantial unevenness in emission distributions across population densities. The lowest 8.2 % of the cumulative population is responsible for 52.6 % of the Scope 1 emissions, contributed by high-emission point sources such as thermal power plants, which are located far from human activity centers. Overall, the spatial difference between Scope 1 and Scope emissions tends to intensify as population density increases (Fig. 5c).

The divergent spatial distributions of Scope 1 and Scope 2 emissions within Shenzhen, as revealed at the landscape scale under the harmonized framework, provide a unique perspective on emission reduction strategies. The grid- and district-level emissions are classified into electricity consumption-led and electricity production-led zones (Fig. 5d). The electricity consumption-led areas underscore the CO_2_ emission responsibility due to high electricity demand despite low local generation, encompassing 84.8 % of grid cells and 8 out of 10 districts in Shenzhen. We illustrate four regions (Fig. 5d) to facilitate targeted emission reduction policies. In Region IV, where Scope 1 emissions exceed Scope 2 emissions, priority should be given to transitioning the energy structure toward clean, renewable resources, possibly supplemented by carbon capture, utilization, and storage techniques. In Region II, where Scope 2 emissions exceed Scope 1 emissions, improving energy efficiency could play a crucial role through green building standards and expanded photovoltaic installations. In Region I, where both types of emissions are high and comparable, a combination of energy production and consumption management strategies is necessary. Targeted emission reduction strategies will help ensure effective and equitable mitigation actions, supporting the carbon reduction efforts of megacities.

## 4 Discussion

### 4.1 Uncertainty evaluation and method limitation

To quantitatively evaluate the uncertainty in Scope 1 CO_2_ emissions across various sectors and fuel types, we employed the Monte Carlo simulation technique, as recommended by the Intergovernmental Panel on Climate Change [43], which has been widely applied in prior research. We assumed that both fossil fuel combustion and EFs followed a normal distribution, conducting 5000 Monte Carlo simulations. The coefficients of variation (CV, defined as the ratio of the standard deviation to the mean) for sector-specific fossil fuel combustion and fuel-specific EFs were extracted from reference values found in the literature [32]. The CV for activity levels was escalated by 50 % relative to the reference values across various sectors to account for the large uncertainties associated with megacity data.

The uncertainty in the total Scope 1 CO_2_ emissions for Shenzhen is 44.8 ± 6.8 Tg CO_2_ (equivalent to ±15.4 %), with a 95 % confidence interval. Stratified by fuel and industry, the agriculture and residential sectors exhibit the highest degree of uncertainty, which is attributed to the greatest variability in the collection of activity-level data; among different fuel types, other gases demonstrate the highest uncertainty (Supplementary Fig. S3). Below, we qualitatively discuss the uncertainties in the spatial distribution of emissions associated with Scope 1 and Scope 2 emissions.

(1) Although we obtained annual energy consumption estimates for individual buildings, uncertainties stemming from variations in occupancy rates, energy efficiency, and actual user behavior may introduce biases in estimating total electricity consumption and the corresponding emissions. By comparing the total simulated building energy consumption with district-level totals from statistical yearbook data (https://tjj.sz.gov.cn), the slope and intercept of the fitting line, as well as the *R*-squared score, were estimated as 0.92, 2.58, and 0.90, respectively (Supplementary Fig. S4). The strong linear correlation between these datasets provides some confidence in the reliability of the building-level energy consumption simulation results.
(2) For industrial combustion emissions, we used publicly available factory-specific NO_*x*_ emissions data in 2013 as a spatial surrogate to downscale industrial CO_2_ emissions. The accuracy of such estimates is affected by delayed reporting caused by enterprise shutdowns, deregistrations, and other activities. We tried to reduce these uncertainties by excluding factories that were reported to have closed between 2013 and 2020 and constraining total emissions based on energy consumption by the industrial sector.
(3) For vehicle emissions, we integrated publicly available traffic flow data in Shenzhen and employed multiday averages to estimate daily traffic flow patterns across various road classes within each district. We constrained vehicle emissions using total fuel consumption data for 2020, proportionally allocated emissions based on traffic flows, and calculated EFs for different vehicle types based on corresponding vehicle speeds to minimize the impact of variability in traffic flow on the emission estimates. However, temporal variations in traffic flow data due to factors such as weather conditions and holidays are not accounted for here.

### 4.2 Future work to enhance landscape emissions monitoring

With the advancement of emerging technologies, particularly the widespread application of big data and artificial intelligence (AI), high spatio-temporal resolution and dynamic, continuous monitoring of urban emissions are expected to show sustained improvement in the future [44,45]. For enhanced dynamic temporal characterization, big data technologies can provide high-frequency, multisource activity datasets (including time-series remote sensing imagery, traffic flow data, electricity consumption metrics, mobility trajectories, and industrial activity records) at monthly, daily, and even hourly temporal resolutions [46], thereby overcoming the limitations of conventional annual-scale accounting methodologies. For long-term historical emission reconstruction and future scenario forecasting, AI algorithms can help address issues with missing statistical data while learning the temporal evolution patterns of emissions from massive time-series datasets. By capturing nonlinear relationships, these approaches enable the building of robust, dynamic models for future emissions prediction, as achieved through long short-term memory networks and transformer architectures [47]. These emerging technologies provide promising potential for responsive carbon mitigation policy assessments at the urban scale, facilitating more timely and adaptive interventions through enhanced feedback mechanisms.

For scientific tools to translate into practical applications in the future, participation from various disciplines and stakeholders is essential, including the collection of data and the integration of technologies. Based on multi-integration, building a measurable, monitorable, and modelable urban emission monitoring system will enable accurate carbon tracking, facilitating the confirmation of emission reduction progress and the implementation of targeted and cost-effective low-carbon policies. Given the common sources of pollutants and greenhouse gases [48], low-carbon and mitigation actions also contribute to improved air quality during urbanization processes. Landscape-level mitigation actions help disaggregate macro-level targets into tangible actions for both policymakers and the public, demonstrating how streets and buildings in residents’ daily lives contribute to climate change [49]. When augmented with AI and digital twin technologies [50]—particularly through interactive dashboards and immersive 3D environments that dynamically visualize the carbon footprint of individual infrastructures and behaviors—these solutions have the transformative potential to reinforce urban carbon reduction efforts.

## 5 Implications

The importance of landscape-level emission monitoring, reporting, and verification solutions has been increasingly recognized [14], with emissions estimates at finer spatial and temporal scales being critical for effective reduction strategies and targeted policies. Nevertheless, challenges remain. Current methods for cities to monitor emissions at high resolution include both bottom–up (emission inventories) and top–down (satellite inversions) methods; however, they differ in terms of accuracy, precision, and scalability. For example, NASA's Orbiting Carbon Observatory-2 and Orbiting Carbon Observatory-3 can characterize CO_2_ surface fluxes at regional and city scales by measuring CO_2_ columns [[51], [52], [53]] based on top–down approaches. Although satellite inversion methods can provide estimates of urban emissions, they cannot trace emissions to their sources or track internal carbon flows. Total emission data alone are insufficient for city decision-makers and community groups, who need to understand sectoral and fuel-specific contributions to urban carbon fluxes [11] and how carbon flows are distributed across consumption behavior [54]. Urban emissions monitoring must reach a high spatial and temporal resolution to trace emissions down to the street or building level, whether through top-down or bottom-up approaches, including point sources from industrial facilities, line sources from street segments, and individual buildings.

At the global scale, there is a universal necessity for a comprehensive emission accounting framework to achieve both effective mitigation and climate justice in urban climate policy, particularly given the widespread reliance on imported electricity in developed regions and megacities. For example, in the United States, the amount of imported electricity in California (70.8 million MWh in 2019) and Virginia (50.1 million MWh in 2023) accounted for 25 % and 36 % of their total electricity consumption, respectively (https://www.eia.gov/todayinenergy/detail.php?id=64104). In France, the Île-de-France region, which includes Paris, imported 66.8 million MWh in 2021 (https://www.statista.com/statistics/1263706/french-regional-electricity-import/). If the emissions related to these large amounts of imported electricity are not considered through the Scope 2 framework, a substantial portion of emissions driven by city demand cannot be effectively monitored and managed. Future applications of a landscape-level carbon accounting framework should not only quantify these implicit Scope 2 emission impacts but also provide granular emission mapping to help trace Scope 1 and 2 carbon spatial and sectoral flows within urban areas. By identifying dominant emission responsibilities across heterogeneous urban districts, this framework will enable effective, equitable, and targeted emission reductions, facilitating the implementation of region-specific strategies, such as clean energy transitions for production-led areas and energy efficiency measures for consumption-led areas.

## CRediT authorship contribution statement

**Yiwen Zhu:** Writing – review & editing, Writing – original draft, Visualization, Methodology, Investigation, Formal analysis. **Yuhang Zhang:** Writing – original draft, Investigation, Formal analysis. **Yi Zhang:** Writing – review & editing, Supervision, Project administration, Conceptualization. **Bo Zheng:** Writing – review & editing, Supervision, Project administration, Funding acquisition, Conceptualization.

## Declaration of competing interest

The authors declare that they have no known competing financial interests or personal relationships that could have appeared to influence the work reported in this paper.
