# Supplementary material for: Tracing CO2 emissions across megacity landscapes: beyond citywide totals to structural heterogeneity and mitigation
Source: Environ Sci Ecotechnol. 2025 Jul 12;27:100602. doi: 10.1016/j.ese.2025.100602 (PMC12284297; doi:10.1016/j.ese.2025.100602)
Supplement: Multimedia component 1 [file mmc1.docx]

**Supplementary Information**

**Tracing CO₂ emissions across megacity landscapes: beyond citywide totals to structural heterogeneity and mitigation**

Yiwen Zhu , Yuhang Zhang, Yi Zhang^*^, Bo Zheng^*^

This PDF file includes:

- Figures S1 to S4
- Table S1

**
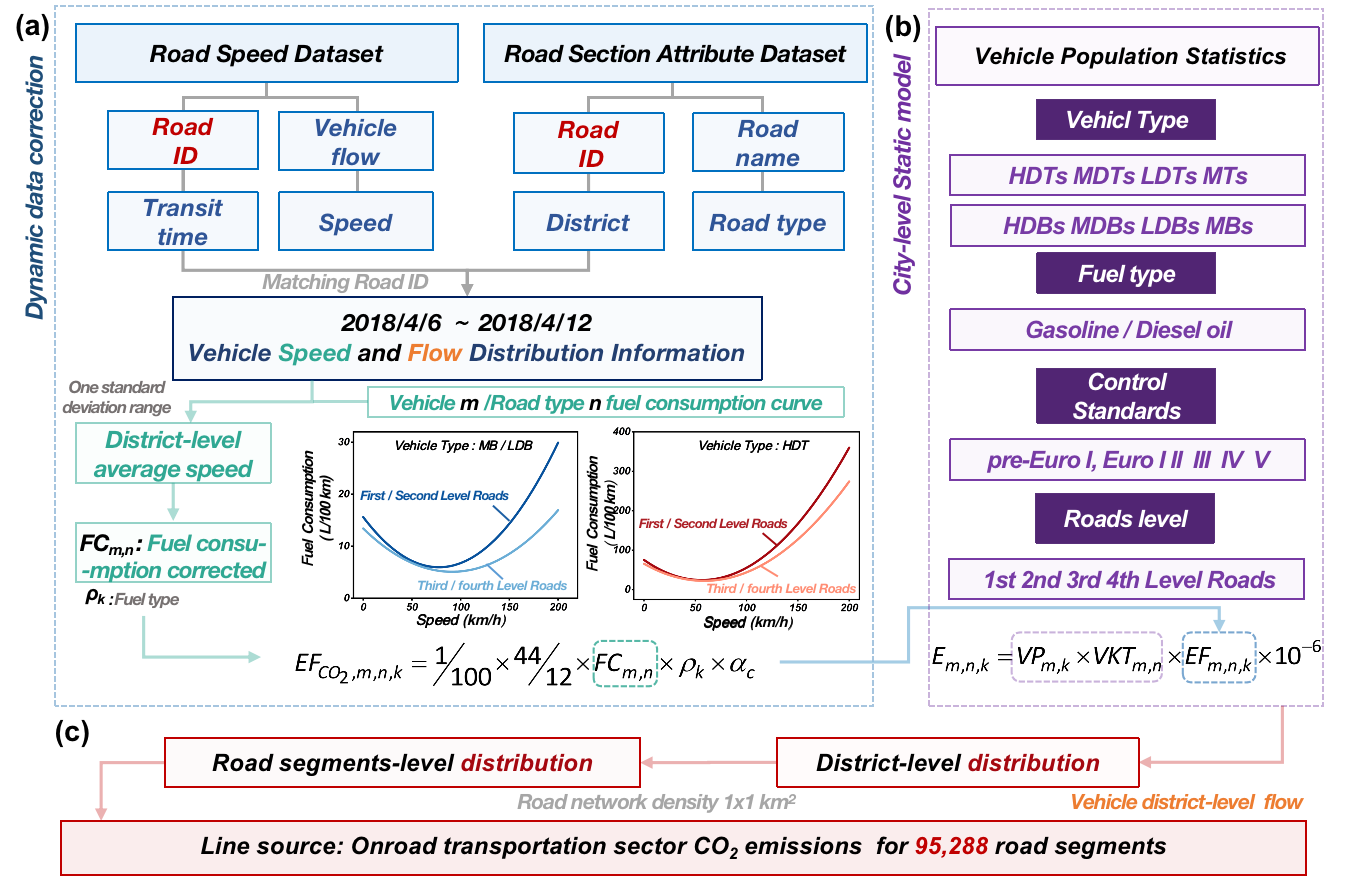
**

**Figure S1. Flowchart of high-resolution city-level vehicle CO_2_ emission mapping method based on dynamic road-level traffic flow data.** (a) The establishment process of the dynamic database and localized emission factor corrections based on average vehicle speed (highlighted in green); (b) City-level vehicle emission model parameters and operation description; (c) Emission spatial distribution based on road-level traffic flow (highlighted in yellow) and road network density.

**
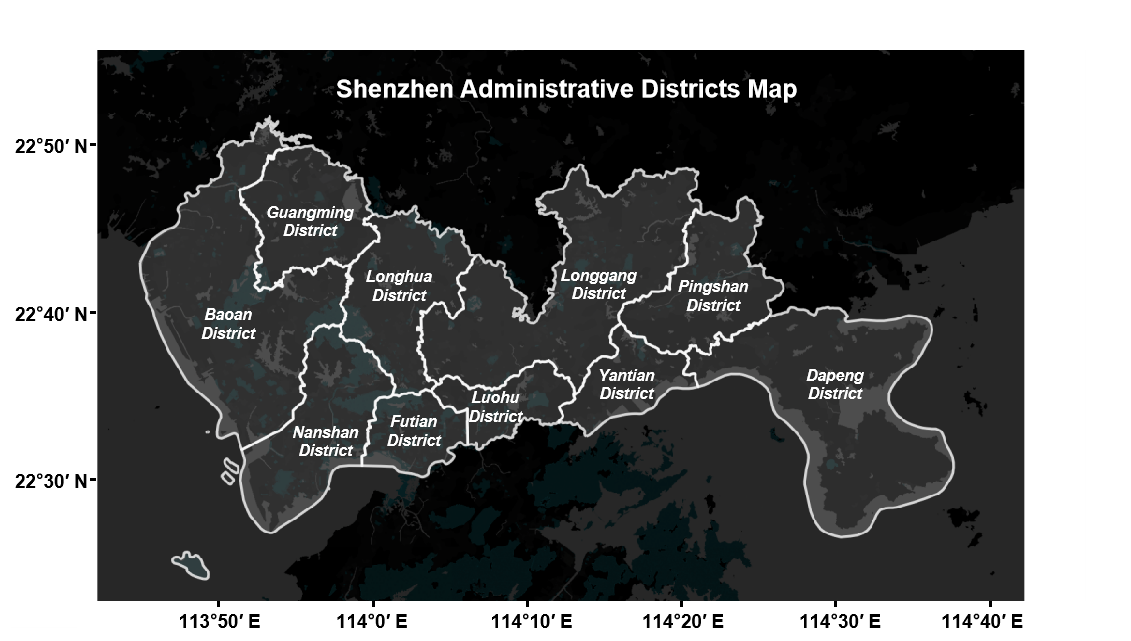
**

**Figure S2. Shenzhen Administrative Districts Map.** Shenzhen has a total of 10 districts, among which Dapeng District is also known as Dapeng New District.


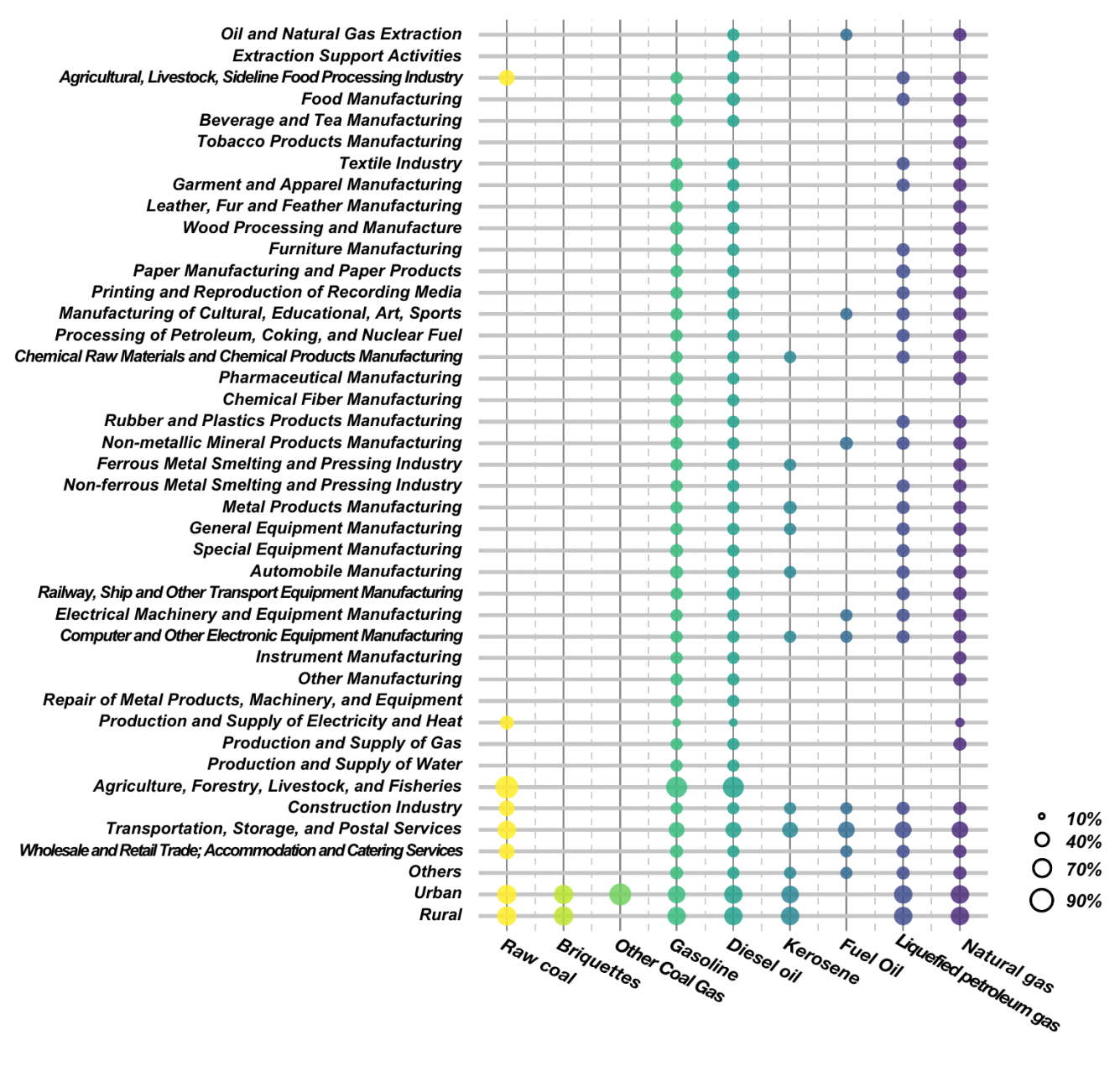
**Figure S3. Monte Carlo simulation of emission uncertainties by industry and by fuel.** The size of the points represents the magnitude of uncertainty. Industries and fuels with no data are those without emission results.

**
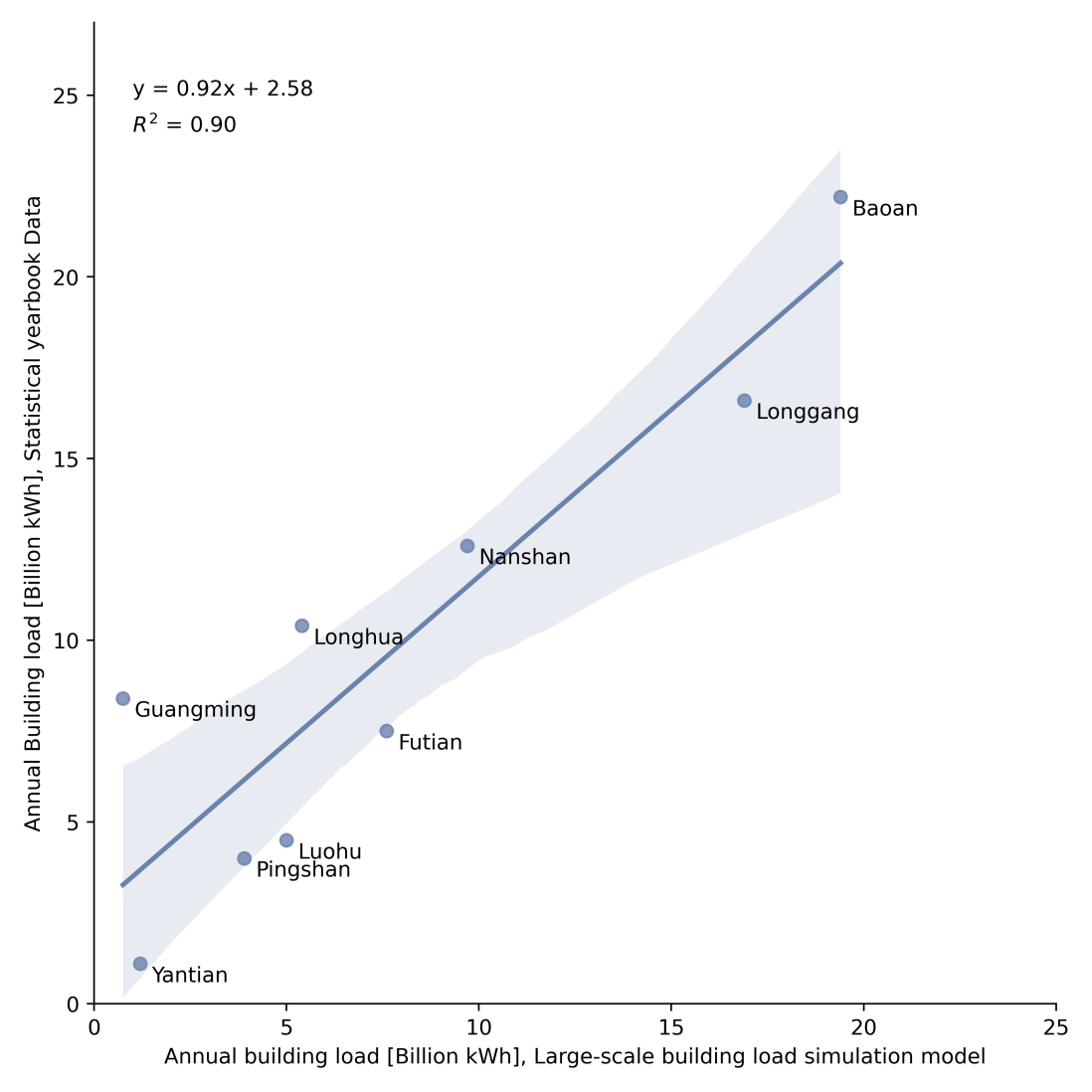
**

**Figure S4. Comparison of the building load simulation results in Shenzhen’s ten districts with electricity consumption data from the statistical yearbook.** The simulated building load was summed up according to administrative divisions before comparison.

|  |  |  | Unit: GgCO_2_ yr^-1^ | | | |
| --- | --- | --- | --- | --- | --- | --- |
| Accounting scope | District Name | Total CO_2_ emissions | Sectoral CO_2_ emissions | | | |
|  |  |  | Point source | Line source | Non-point source | Other sources |
| **Scope 1** | Baoan District | 3887.3 | 573.6 | 2077.9 | 1234.5 | 1.3 |
|  | Nanshan District | 19781.7 | 17239.5 | 1681.4 | 857.9 | 2.9 |
|  | Guangming District | 1839.6 | 83.9 | 1249.4 | 501.9 | 4.5 |
|  | Longhua District | 3375.9 | 770.2 | 2035.2 | 569.3 | 1.1 |
|  | Futian District | 3315.8 | 689.4 | 2213.5 | 409.4 | 3.5 |
|  | Luohu District | 2758.6 | 12.1 | 2511.1 | 233.2 | 2.1 |
|  | Longgang District | 3438.5 | 593.5 | 1970.2 | 872.3 | 2.5 |
|  | Yantian District | 1369.3 | 20.8 | 1288.7 | 59.4 | 0.4 |
|  | Pingshan District | 1525.9 | 93.4 | 1207.5 | 222.9 | 2.1 |
|  | Dapeng District | 3524.4 | 2328.6 | 1186.2 | 7.1 | 2.5 |
| **Scope 2** | Baoan District | 10741.2 | 573.6 | 2077.9 | 8088.5 | 1.3 |
|  | Nanshan District | 8153.4 | 848.1 | 1681.4 | 5620.9 | 2.9 |
|  | Guangming District | 4625.8 | 83.9 | 1249.4 | 3288.1 | 4.5 |
|  | Longhua District | 5838.6 | 72.0 | 2035.2 | 3730.3 | 1.1 |
|  | Futian District | 4917.8 | 18.3 | 2213.5 | 2682.5 | 3.5 |
|  | Luohu District | 4053.4 | 12.1 | 2511.1 | 1528.0 | 2.1 |
|  | Longgang District | 7816.6 | 128.6 | 1970.2 | 5715.2 | 2.5 |
|  | Yantian District | 1699.0 | 20.8 | 1288.7 | 389.1 | 0.4 |
|  | Pingshan District | 2763.5 | 93.4 | 1207.5 | 1460.5 | 2.1 |
|  | Dapeng District | 1350.9 | 115.5 | 1186.2 | 46.7 | 2.5 |

**Table S1 . The CO_2_ emissions by sector in the ten districts of Shenzhen in 2020**
